# Application of the 50% Hydrazine Solution Method for O-Glycans Release, their Chemical Labeling, and HPLC Separation

**DOI:** 10.1080/15376510701623755

**Published:** 2008-06-23

**Authors:** D. G. Kisiel, I. Radziejewska, A. Gindzieński

**Affiliations:** Department of Medical Chemistry, Medical University of Białystok Mickiewicza, Białystok, Poland

**Keywords:** ABEE, Chemical Labeling, Mucin, O-Glycans, UV Detection HPLC

## Abstract

Mucins are high molecular mass glycoproteins with oligosaccharides O-bonded to the protein core. β-elimination is the most popular method used for releasing of O-glycans. However to such released glycoforms it is difficult to introduce a label to amplify a signal for oligosaccharide detection.

In our study we used a combination of the β-elimination and hydrazinolysis methods. Released glycoforms were labeled with para-amino benzooic acid ethyl ester (ABBE) and fractionated on HPLC column.

This combined procedure seems to be a good tool for O-glycans analysis.

## INTRODUCTION

O-glycoproteins are widely distributed in nature and most of them are known as mucins. One of the important functions of mucins is that they are the first line of defense against environmental pathogens and toxins, and that they form tissue antigenic determinants. Their carbohydrate portion is mostly composed of O-bonded oligosaccharides, in some instances tightly covering protein backbone. The structure, antigenic properties, and interactions of oligosaccharides with other particles and cells are important for understanding of body function and its pathology.

There are many published methods for releasing and labeling N-glycans, including enzymatic methods. For O-glycans, where enzymatic methods are not applicable, the most widely used method is alkaline β-elimination with sodium borohydride reduction of freshly restored carbonyl (Carlson 1968; Kotani and Takasaki 1997). As a final product oligosaccharide alditols are obtained. In such form, it is difficult to introduce a label to amplify a signal for oligosaccharide detection. Thus, the most valuable methods for O-glycan analysis should offer glycans release in their reducing form. There are some methods described for O-glycan release with anhydrous hydrazine (Patel et al. 1993; Merry et al. 2002).

In the present investigations we used a combination of the β-elimination and hydrazinolysis methods (Cooper et al. 1994). Oligosaccharides were released in the alkaline milieu of both thriethylamine and hydrazine in one water solution. After deblocking of N-acetylgalactosoamine hydrazones, the restored oligosaccharide carbonyl groups were labelled with para-amino benzoic acid ethyl ester (ABEE) (Matsuura and Imaoka 1988; Kisiel et al. 1999) and oligosaccharides were fractionated on HPLC columns with UV detection. The chromophore group introduced to the oligosaccharide allows the detection in common UV monitors. In such modification, this combined procedure becomes a very good tool for structural analysis of O-linked oligosaccharides, usually very complicated and equipment consuming.

## MATERIALS AND METHODS

### Preparation and Purification of Glycopeptides

Pig gastric mucin was isolated from pig stomachs as described previously (Gindzieński and Zwierz 1987) but somewhat modified. Briefly, the mucin was purified by gel exclusion chromatography on Bio-Gel A 15 m column (4.2 x 40 cm) in 0.05 M borate buffer (pH 7.0) containing 6 M urea, dialyzed against water for 48 h, and lyophilized. Such material was digested with Pronase (Boehringer Mannheim) according to the company-included protocol, and the obtained glycopeptide pool was isolated on Bio-Gel A 0.5 m column (2.5 x 80 cm) with the same buffer as an eluent. The eluate was monitored for neutral carbohydrates with the phenol-sulphuric acid method (Dubois et al. 1956). Glycopeptide-containing Vo peak was collected, dialyzed, and lyophilized. Monosaccharide composition of the glycopeptide preparation was determined by capillary gas chromatography as silyl ethers of methyl glycosides (Chaplin 1982) in modification (Roberton et al. 1991). For further analysis, the glycopeptide pool was solubilized in 50% pyridine solution and divided for several aliquots (5 mg), each containing 625 μg of neutral carbohydrate. Aliquots were lyophilized and stored dry at −60°C.

### Liberation of Glycans

In the hydrazine method, each glycopeptide sample (5 mg; each containing 625 μg of neutral carbohydrates) was heated in a sealed glass vial at 45°C in 2 mL of 50% hydrazine solution containing 0.2 M triethylamine, for different periods of time: 14, 24, 36, 48, 60, and 72 h. After evaporation of the postreaction volatile components, the dry sample was dissolved in 6.4 mL of saturated NaHCO_3_ solution and 0.32 mL of acetic anhydride was added. After 20 min of incubation, the solution was passed thorough the Dowex 50 WX8 column (10 mL, H^+^ form) and the water eluate was concentrated on a rotary evaporator. In three methods (i.e., classic alkali β-elimination with NaBH_4_ as a reducing agent; method with TEA and 50% hydrazine; and a mix of both methods, that is NaOH and 50% hydrazine), the remaining polypeptide was separated off using centrifuge Centricon 10 separators (4000 RPM for 2 h) and the undermembrane fraction was treated as the oligosaccharide pool. Recovery of oligosaccharide hydrazones and oligosaccharide polyols was determined by the phenol-sulphuric acid method (Dubois et al. 1956). Oligosaccharide hydrazones were converted into reducing oligosaccharides with 20% of acetone solution (24 h at 55°C in 2.0 mL of the final volume) and volatile products were evaporated.

### Labeling of Glycans Pool

Mucin oligosaccharides obtained from the hydrazine method were submitted to reductive amination with ABEE (Matsuura and Imaoka 1988). To obtain an optimal molar ratio of reducing glycans to the labeling reagent (1:40), prior to labeling, an amount of N-acetylgalactosamine in the oligosaccharide sample was determined. To the dried samples in the reaction vials 10 μL of water was added, followed by 40 μL of freshly prepared labeling reagent (35 mg of ABEE, 3.5 mg NaBH_3_CN, 350 μL of methanol, and 41 μL of glacial acetic acid). Vials were tightly closed and heated at 80°C for 1 h, with occasional mixing. Then 1 mL of water was added and the ABEE excess was removed using five times repeated extraction with 1 mL of diethyl ether. Excess of the ABEE in the water phase was removed by solid-phase extraction using 3 mL silica LC-18 SPE columns (Supelco), and the UV-absorbing labeled oligosaccharides were eluted with water. For column calibration a similar labeling procedure was performed with isomalto-oligosaccharides of dextran hydrolysate and the labeled oligosaccharides were purified using the LC-18 columns eluted with 10% solution of acetonitrile in water.

### HPLC Separation of Labeled Glycans

ABEE-oligosaccharides were chromatographed on a Cosmo-gel DEAE anion exchange HPLC column (7.5 x 75 mm, Nacalai Tesque Inc.) using Knauer WellChrom HPLC Pump K-1001 and Model 87.00 UV Knauer absorbance detector. The column was pre-equilibrated with 0.5 mM of sodium acetate solution and eluted with a linear gradient of sodium acetate (0.5 mM to 150 mM for 30 min with 1 mL/min flow rate).

Peak material obtained from several separations was collected, pooled, concentrated by evaporation, and desalted with the LC-18 SPE columns eluted with water and then with 10% of acetonitrile. The concentrated material of each peak was subjected to chromatography on a LiChrospher-100 NH_2_ 5 μm column (8 x 250 mm, Knauer). The column was eluted at ambient temperature in the gradient mode at 2.0 mL/min flow rate, using acetonitrile and water. Prior to analysis the column was pre-equilibrated with 80:20 (v/v) acetonitrile:water mixture. After injection of the sample, the elution was carried out for 30 min with a linear gradient of acetonitrile until acetonitrile:water ratio reached 65:35, then for the next 30 min until a 60:40 ratio, followed by 10 min to reach a 50:50 acetonitrile:water ratio.

## RESULTS

Isolated mucin glycopeptide was submitted to gas chromatographic analysis for its monosaccharide composition. Results presented in Table 1 show that composition of glycopeptide oligosaccharides is typical for mucin. Sialic acid was not determined. GalNAc originates from the reducing as well as nonreducing end.

**TABLE 1:** Monosaccharide composition of pig gastric mucin glycopeptide preparation

Monosaccharide	Fucose	Mannose	Galactose	GalNAc	GlcNAc
Amount of monosaccharide (μmol)	0.146	0.008	0.271	0.100	0.279
Approximate mol ratio to GalNAc	1.5	0.1	2.7	1	2.8
Mass percentage (%)	15	1	31	14	39

A sample of isolated glycopeptide was submitted to methanolysis and reacetylation followed by silylation, and analyzed for its monosaccharide composition by gas chromatography.

Solubilization of dry glycopeptide pool is not recommended in buffers containing SDS or urea due to difficulties in removing those substances affecting later contamination of the tested sample. Urea and SDS interfere in GC and in colorimetric methods. Fifty percent pyridine solution was chosen to homogenize the glycopeptide pools from different separations. Ten percent, 25%, 50%, and 75% concentrations of pyridine or methanol solutions were tested and 50% solution was deemed as satisfying in solubilization of dry material. Moreover, pyridine does not interfere in other assays and evaporates easily.

To determine an optimal period of time for pig gastric mucin oligosaccharide release and recovery for the method with NaOH/NaBH_4_ as well as the method with 50% hydrazine/TEA, six aliquots of standard glycopeptide were submitted to a releasing procedure for 14, 24, 36, 48, 60, and 72 h. An optimal time for both methods, measured in neutral carbohydrates recovery, was found to be 48 h (data not shown).

Table 2 presents the results of neutral carbohydrate determinations in samples, where releasing was carried out in two methods and modification of those with NaOH and 50% hydrazine. The first recovery line represents the level of destruction in general for three methods (part of oligosaccharides that “survived” elimination reaction), while the second recovery line shows what part of oligosaccharides in reacted pool was released indeed. The third recovery line recapitulates the whole procedure of oligosaccharide releasing.

**TABLE 2:** Comparison of neutral carbohydrate recovery after subsequent steps of oligosaccharide isolation

	TEA/N_2_H_4_	NaOH/NaBH_4_	NaOH/N_2_H_4_
1. Content of neutral carbohydrates in starting glycopeptide (μg)	625	625	625
2. Content of neutral carbohydrates in β-elimination products (μg)	510	350	260
Recovery of neutral carbohydrates after β-elimination (2:1)	81.5%	56%	41.5%
3. Content of neutral carbohydrates in undermembrane fraction (μg)	300	281	190
Recovery of oligosaccharides from β-elimination products (3:2)	59%	80%	73%
Recovery of oligosaccharides from starting glycopeptide (3:1)	48%	45%	30%

Standardized samples of glycopeptide were submitted to oligosaccharide releasing procedure according to the hydrazine method (TEA/N_2_H_4_), to the classical reductive β-elimination (NaOH/NaBH_4_), and to a modified hydrazine procedure with NaOH instead of 3-ethylamine (NaOH/N_2_H_4_). Neutral carbohydrates were determined according to the method of Dubois et al. (1966).

**FIGURE 1:**
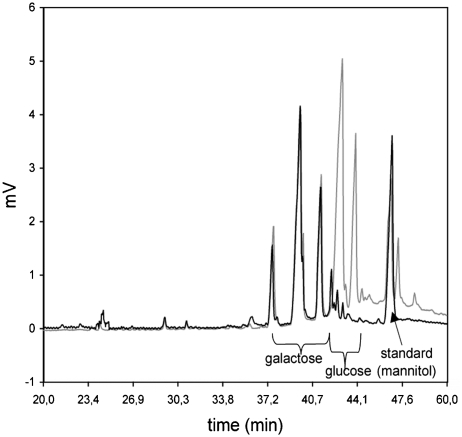
Gas chromatographic separation of lactose constituents. The gray line represents three peaks of galactose and two peaks of glucose, all of untagged lactose origin, and internal standard, mannitol. The black line represents lactose after labeling with p-aminobenzoic acid ethyl ester (ABEE).

Lack of an ABEE-labeled monosaccharide from the reducing end of oligosaccharide in the gas chromatographic carbohydrate determination is a tremendous merit of this method. This was confirmed with ABEE-labeled lactose, where no glucose is present on the chromatogram (Fig. 1).

**FIGURE 2:**
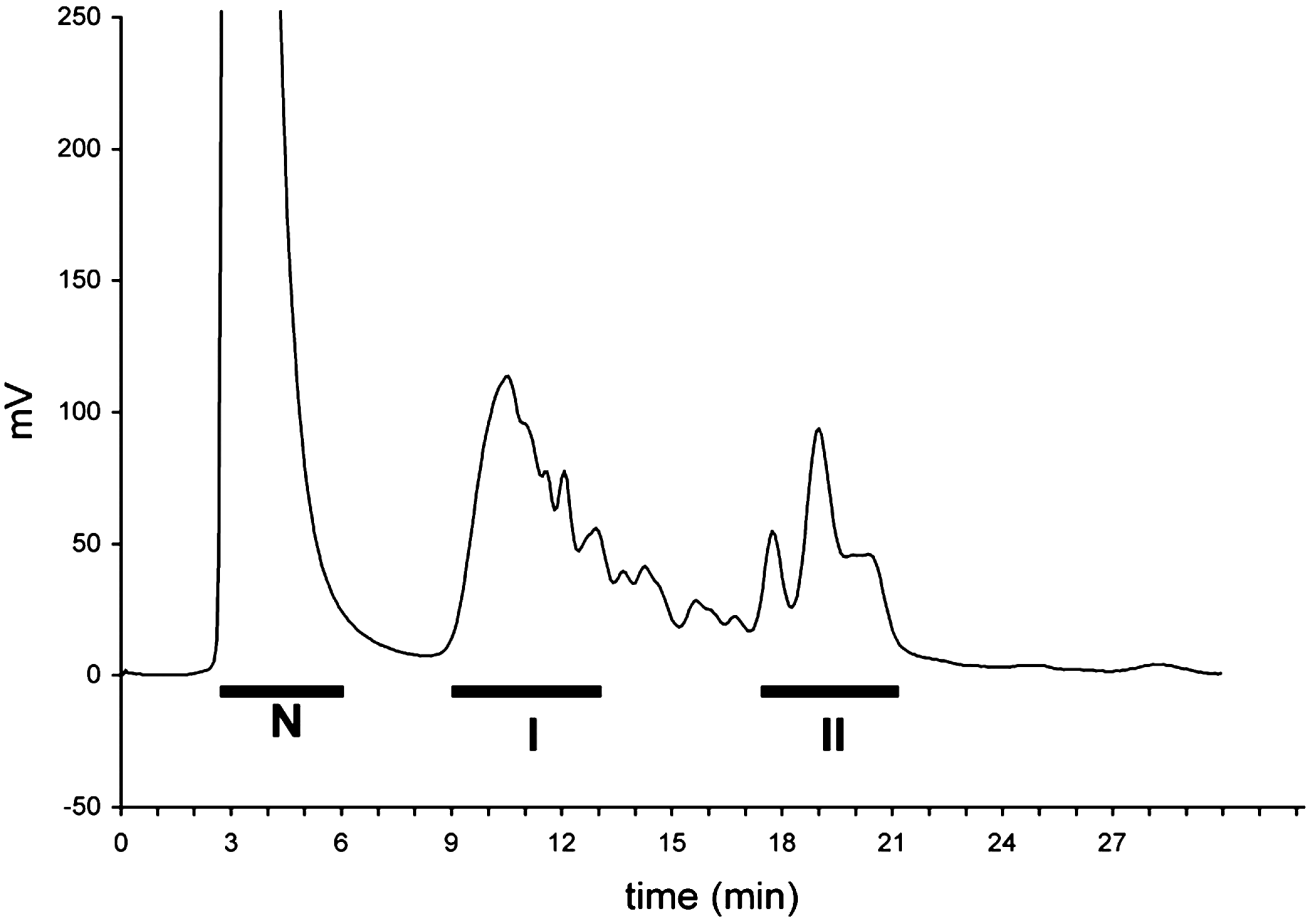
HPLC separation of ABEE-labeled mucin oligosaccharides on a DEAE column. N, neutral oligosaccharide fraction; I and II, acidic oligosaccharides. Neutral oligosaccharides were pooled, desalted on LC18 SPE cartridges, condensed, and submitted to rechromatography on amine phase column.

**FIGURE 3:**
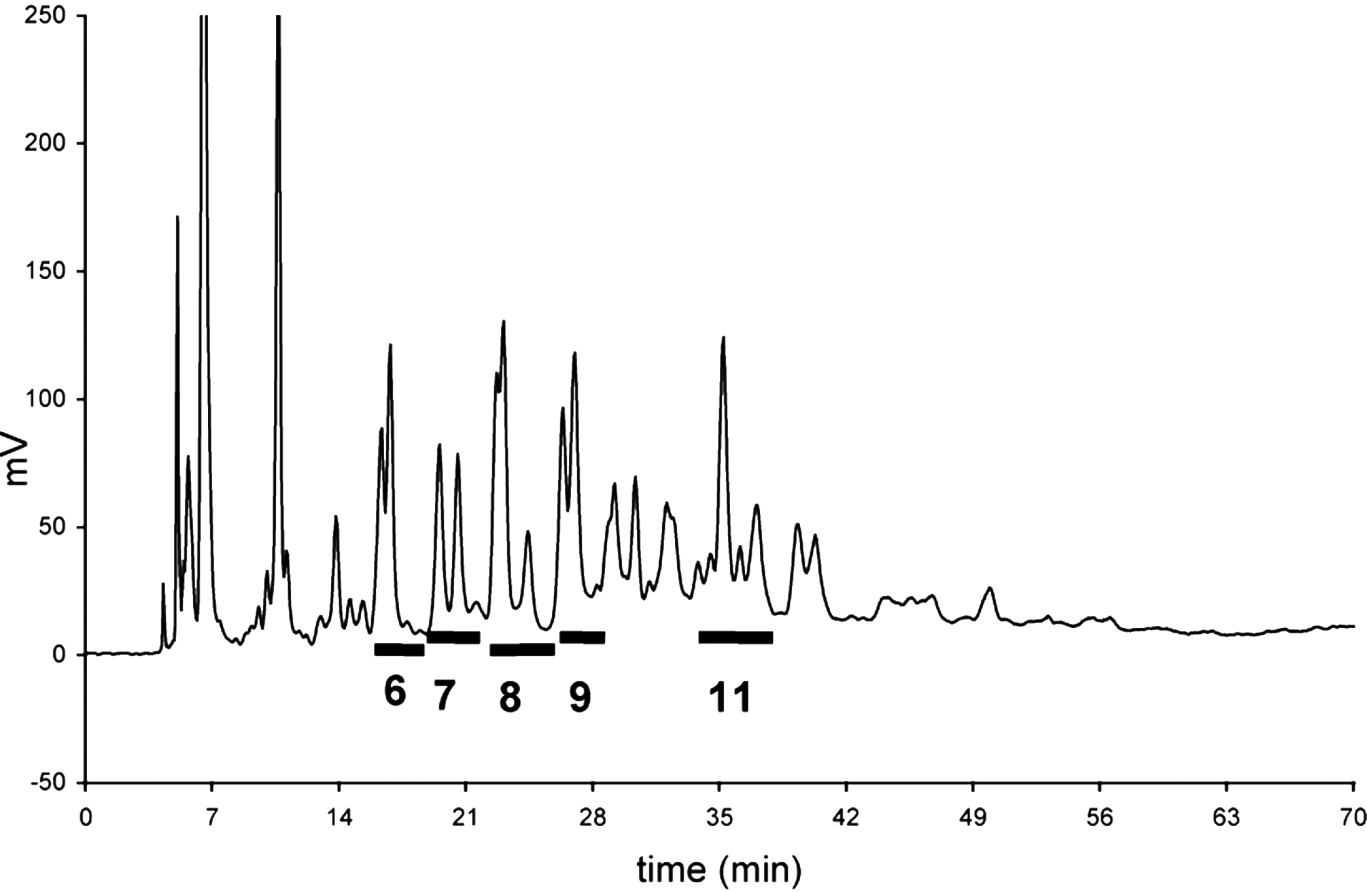
Separation of ABEE-labeled neutral oligosaccharides from pig gastric mucin on LiChrospher 100 NH_2_ HPLC column. Numbered peaks were analyzed for their monosaccharide composition (Table 3).

Labeled, mucin-originated glycans were separated on DEAE column and three fractions were eluted according to their increasing acidity (Fig. 2). The concentrated neutral pool of oligosaccharides was fractionated on a LiChrospher NH2 HPLC column and 13 well-defined chromatographic peaks were obtained (Fig. 3). Some of these peaks were examined for their monosaccharide composition by gas chromatography, of which results are presented in Table 3.

**TABLE 3:** Monosaccharide analysis of peaks eluted from the LiChrospher NH_2_ column

	Contents of each monosaccharide in each peak (μmol/peak)				
	
Number of peaks	Fuc	Gal	Glc	GalNAc	GlcNAc
6	0.045	0.074	0.002	0.005	0.065
7	0.055	0.063	0.025	0.005	0.050
8	0.062	0.051	—	Trace	0.031
9	0.016	0.030	0.004	Trace	0.011
11	0.020	0.041	0.008	0.005	0.043

Released monosaccharides were fractionated on a DEAE column and the neutral oligosaccharide pool was fractionated on the LiChrospher NH_2_ column. Some of the obtained peaks from the LiChrospher column were pooled and submitted to gas chromatographic analysis for their monosaccharide composition. Fuc, fucose; Gal, galactose; GalNAc, N-acetylgalactosamine; GlcNAc, N-acetylglucosamine.

## DISCUSSION

There are many sophisticated techniques used for O-glycan analysis (Peter-Katalinic 2005) like HPAEC, NMR, or MALDI-TOF. We combined and elaborated a technique that allows us to analyze O-linked oligosaccharides on simple and popular HPLC systems with UV detector or (after use of appropriate tagging substance) fluorescence detector. Any such application was noted in scientific literature. It simplifies and accelerates research of O-linked oligosaccharides and the final product is ready for labeling due to a free semiacetal, reducing group on C1 of core GalNAc. Other more often used methods, like classic β-elimination with alkali (Carlson 1968; Kotani et al. 1997) or hydrazinolysis in anhydrous conditions (Patel et al. 1993; Merry et al. 2002), are not suitable if the final product must be tagged without radioisotope or one of the reactant needs additional preparation and is highly dangerous and toxic (anhydrous hydrazine).

Results of analysis of oligosaccharides of mucin glycopeptide for its monosaccharide composition presented in Table 1 prove composition typical for mucin with lactosoaminyl residues on the nonreducing end. As can be deduced from Table 2, the aqueous hydrazine solution method is less destructive for neutral carbohydrates than the classical β-elimination one; however, this method is less effective in oligosaccharide recovery. For the hydrazine method, the percentage of oligosaccharide released from pig gastric mucin glycopeptide is somewhat higher than with the classical method. With the modified hydrazine method, in which triethylamine was replaced with 0.05 M NaOH, the level of neutral carbohydrates loss reaches over 70%. Method with hydrazine is less destructive (81.5% recovery of saccharides) but not complete (48% final recovery comparable with 45% in classic β-elimination). It shows that this method is very competitive and additionally has one more advantage. The aqueous hydrazine solution method results in oligosaccharides with terminal monosaccharide reducing carbon C1, which can be labeled with a wide spectrum of labeling substances. In this paper we use UV-absorbing compound ABEE, elaborated for labeling of N-glycans (Matsumoto et al. 2000; Matsuura et al. 1992), mostly released with the enzymic methods. This type of labeling is highly specific to the carbonyl group and results seem to be more reliable for O-linked oligosaccharide analysis than the classical β-elimination and radiolabeling with borotritide.

Another merit of this method is lack of an ABEE-labeled monosaccharide from the reducing end presence of oligosaccharide in the gas chromatographic carbohydrate determination. This was confirmed with ABEE-labeled lactose, where no glucose is present on the chromatogram (Fig. 1). According to this observation, in the analysis of mucin origin-labeled oligosaccharides, the presence of N-acetylgalactosamine on the chromatogram can be interpreted as originating from the nonreducing part of the oligosaccharide chain only. This can also be helpful for measuring the size of the unwanted oligosaccharide “peeling” reaction (Chamow and Hedrick 1988), in order to calculate the diminution of the amount of monosaccharides other than protein O-linked GalNAc in the gas chromatographic analysis. The aqueous hydrazine solution method is an alternative approach to the O-glycan releasing process, combining the β-elimination mechanism with immediate protection of the released glycans from the “peeling” reaction in their hydrazone form. The reducing oligosaccharides can be easily labeled with any marker for chromatographic purposes.

The use of anhydrous hydrazine for the same is more complicated, as this reagent is not available from reagent companies and the process of drying its water solution needs special laboratory conditions. In comparison to the alkaline borohydride elimination, the aqueous hydrazine solution method offers similar effectiveness and simple, very specific to carbonyl group labeling. The high sensitivity of detection of such derivatives makes analysis feasible to microgram level of naturally occurring glycoproteins.

## ABBREVIATIONS

GalNAc: N-acetylgalactosamine

GlcNAc: N-acetylglucosamine;

ABEE: *p*-aminobenzoic acid ethyl ester
